# COVID-19 pandemic- knowledge, perception, anxiety and depression among frontline doctors of Pakistan

**DOI:** 10.1186/s12888-020-02864-x

**Published:** 2020-09-23

**Authors:** Faridah Amin, Salman Sharif, Rabeeya Saeed, Noureen Durrani, Daniyal Jilani

**Affiliations:** Liaquat National Hospital and Medical College, Karachi, Pakistan

**Keywords:** COVID-19, Pakistan, Frontline physicians, Anxiety, Depression, Knowledge, Perception

## Abstract

**Background:**

COVID-19 is a global pandemic and has become a major public health burden worldwide. With already fragile healthcare systems it can have long lasting effects in developing countries. Outbreaks especially a pandemic situation evokes fear related behaviors among healthcare professionals and there is always an increased risk of mental health disorders. Therefore, this study aims to determine knowledge and perception about this pandemic, prevalence and factors associated with anxiety/depression among frontline physicians of Pakistan.

**Methods:**

Data were collected through an online survey released in the last week of March-2020. 389 frontline physicians from all four provinces and 65 cities of Pakistan participated. Survey questionnaire consisted of 4 parts including informed consent section, demographic section, knowledge and perception about COVID-19 pandemic and assessment of depression through World Health Organization Self-reporting questionnaire (SRQ-20). A score of 8 or above on SRQ-20 was used as cut-off to label the participant as depressed. Data was analyzed using SPSS version22.

**Results:**

A 43% prevalence of anxiety/depression among frontline physicians of Pakistan was reported. Almost all the doctors had moderate to high knowledge score. Majority of participants marked N-95 mask as “essential” during aerosol generating procedures, assessing patients with respiratory symptoms, in COVID patient-care area, ER triage and direct care of COVID-19 patient. Only 12% of the doctors were fully satisfied with the provision of PPEs and almost 94% felt unprotected.

In multivariable model, assessing more than five COVID suspects/day (aOR = 2.73, 95% CI: 1.65–4.52), working 20 h/week or less (aOR = 2.11, 1.27–3.49), having children among household members (aOR = 1.58, 95% CI: 1.00–2.50) and moderate to low knowledge of the infection (aOR = 2.69, 95% CI: 1.68–4.31) were found to be independent predictors of anxiety/depression among physicians.

**Conclusion:**

Anxiety/depression among more than a third of frontline doctors of Pakistan warrants the need to address mental health of doctors caring for patients during this pandemic; control modifiable factors associated with it and explore the effectiveness of interventions to promote psychological well-being of physicians.

## Background

Severe Acute Respiratory Syndrome Corona Virus 2 disease (SARS-CoV-2; previously named 2019 novel corona virus) or COVID-19 is a global pandemic and has become a major public health burden worldwide. First reported as cases of pneumonia of unknown etiology in Wuhan, China on 31st December 2019, the epidemic was associated with seafood exposure in one of the markets in Wuhan and later identified as a new strain of Corona virus [1]. Within a month, a rapid wave of infection affected more than two hundred countries of the world outside China [2]. By the end of April, the cases exponentially increased to affect more than 3 million population with more than 200,000 deaths attributable to COVID-19 globally [3]. Overwhelming COVID-19 outbreaks have occurred in the developed countries including USA and Europe resulting in overburdening of their health care system [4]. With already fragile healthcare systems; the pandemic can have devastating and long lasting effects in developing countries.

In Pakistan, the first imported case of COVID − 19 was reported on 26th February 2020 and just in a few months by the end of April 2020, the numbers are approaching more than 20,000 cases with a death rate of around 2% [3]. Though, relatively lower in Pakistan, the high mortality rate in other countries like Italy, Iran and USA [3] is alarming and provokes xenophobia among healthcare workers and the general public at large.

Outbreaks especially a pandemic situation evokes fear related behaviors among people and there is always an increased risk of mental health disorders [5]. As reported in previous studies during Ebola outbreak in Sierra Leone, 48% of general population reported symptoms of depression and anxiety [6]. Healthcare workers including front line physicians, nurses and paramedical staff dealing with this situation at forefront were especially vulnerable [7, 8]. A recent study in China reported a very high prevalence of depression (50.4%), anxiety (44.6%) and insomnia (34%) among health care workers directly involved in care of COVID-19 patients [9]. Various factors hypothetically account for an increased incidence of anxiety and depression among frontline physicians and general practitioners in Pakistan and other developing countries. One of the plausible factors could be perception of an inadequate capacity of healthcare system to handle this outbreak while having witnessed collapse of best healthcare systems even in the developed world. Living in joint family system, Pakistani physicians may also be more concerned about getting infected and transmitting infections to their household members due to suboptimal infection control practices at their workplaces whereas social isolation may also aggravate stress and lead to psychosocial illness [10, 11]. Injudicious use of personal protective equipments (PPEs) by common people leading to its shortage for protection of frontline workers is yet another anxiety provoking factor among healthcare workers. Moreover, in this era of information technology while people are getting quick updates on the evolving situation through various media, this may act as a double edged sword to increase their anxiety regarding the spread of disease and mortality among healthcare workers particularly physicians.

Physicians and other healthcare workers being considered as the heroes of this pandemic situation are the main force on which foundation of any healthcare system rests; hence it is of utmost importance that their mental and physical wellbeing must be taken care of so that they can perform their duties in the most efficient manner. Scarce data indicates one third of the physicians suffering from anxiety and depression in Pakistan even before the pandemic [12]. Yet, because of the novelty and rapid evolution of this crisis situation there are no researches available, especially in developing countries to determine the impact of these hypothesized factors on the mental health of frontline physicians. Hence it is important to conduct a study to evaluate the knowledge and perception about this pandemic, frequency of and factors associated with depression and anxiety among frontline physicians and general practitioners working in private and public sector healthcare institutions during COVID-19 pandemic in Pakistan. The findings of this study will help policy makers and healthcare administrators to design protocols, policies and interventions to promote mental wellbeing of frontline workers at their work place. This study will provide basis for further analytical studies to assess the impact of various interventions on addressing mental health problems among health care workers in the current pandemic situation.

## Methods

### Study design, duration and setting

This is a cross-sectional study. Data were collected through an online survey released in the last week of March, 2020 and closed when the sample size was achieved in the last week of April, 2020. The survey was forwarded to potential participants through physician associations, Pakistani physician groups on social media, young graduates and physician societies of Pakistan. Online consent was taken and eligibility criteria were checked before data entry. All physicians working in emergency or primary care services, outpatient department, wards, special care units or intensive care units where COVID suspects, confirmed cases and patients with respiratory illnesses are provided consultation or inpatient care were selected. Doctors who were not involved directly with patient care such as basic scientists, university or college lecturers and those who already had a history of mental disorder were excluded.

### Sample size

NCSS PASS software version 11 was used for sample size calculation. A recent study among healthcare workers involved in care of COVID-19 patients showed that the frequency of anxiety and depression among males and females was significantly different (38.75 and 45.95% respectively) [9]. Therefore with 5% probability of type 1 error and power of 80%, with an odds ratio worth detecting of 2.0, sample size was calculated as at least 385 participants.

### Survey questionnaire

An online questionnaire was designed on Google app; the first part of which was informed consent. Socio-demographic factors were recorded in the second part while the third part of questionnaire consisted of questions to assess COVID-19 knowledge and perception about the need of different PPEs in various clinical settings. Seven questions were asked to assess knowledge about rate of complications and mortality rate in Pakistan, high risk groups, yield of different samples and diagnostic tests for COVID-19. Total knowledge score was 7 and a score of 6–7 was considered as “high”, 4–5 as “moderate” while less than 5 as “low” knowledge score. Questions related to knowledge are provided in Additional file 1.

Anxiety/depression was screened based on a validated World Health Organization Self Reporting Questionnaire (SRQ-20) [13]. A score of 8 or above was considered as a positive case.

### Data analysis

Data were transferred from excel to SPSS version 22 and analyzed. Frequencies and proportions for categorical variables were computed. Continuous variables (age) was expressed as median (IQR: inter-quartile range) after assessing assumption of normality through Shapiro-Wilk test. Univariable logistic regression was performed to explore association of each independent variable with the outcome (anxiety/depression). Multivariable logistic regression was used to measure the association of multiple independent variables with the outcome (anxiety/depression) by computing adjusted odd ratios and their 95% confidence interval. Variables with *p*-value < 0.2 in univariable analysis were subsequently included in the final multivariable model [14]. Final effect model was made by backward likelihood ratio elimination method. Statistical significance of independent variables in the multivariable model was considered at p-value < 0.05.

## Results

### Characteristics of study participants

A total of 389 complete surveys were received from eligible participants. Frontline physicians from 65 cities of Pakistan participated. Majority of the responses were from Karachi (*n* = 132, 33.9%) where highest number of COVID cases have been reported so far [3]. Out of 389 respondents, 289 (74.3%) were interns or post graduate trainees (both groups referred as trainees) while 100 (25.7%) were consultants with a post graduate qualification. Male and female respondents were 201 (51.7%) and 188 (48.3%) respectively. The overall median age of respondents was 35 (IQR = 30–45) years while median age for trainees and consultants was 34 (IQR = 30–40) and 44 (IQR = 38–51) years respectively. More than half of the physicians who responded were from public sector healthcare institution (*n* = 208, 53.5%). Responses were received from physicians who were working in emergency service (*n* = 147, 37.8%), out-patient department (OPD) (*n* = 153, 39.3%) and wards (*n* = 31, 8%) whereas 58 (14.9%) were working in other healthcare units such as high dependency and intensive care units. Among provinces of Pakistan, majority of physicians responded from Sindh (*n* = 170, 43.7%) and Punjab (*n* = 130, 33.4) while there were a few from Khyber Pakhtunkhwa (*n* = 69, 23.9%), Azad Kashmir (*n* = 2, 0.5%) and Balochistan (*n* = 1, 0.3%).

### COVID-19 related knowledge

On inquiring about the groups at higher risk of complications, 96.1% of the participants responded that elderly are more at risk of complications while 94.3% reported that immuno-compromised patients may also exhibit a high rate of complication. Interestingly, half of them also believed that adults (*n* = 161, 41.4%) and children (*n* = 215, 55.3%) are among high risk groups. Majority (88.2%) correctly responded to the current mortality rate of COVID-19 in Pakistan.

Majority of the participants identified that broncho-alveolar lavage and nasopharyngeal swab have a higher yield for virus (*n* = 367, 94.34%). Polymerase chain reaction (PCR) was correctly identified as the standard diagnostic test for COVID-19 by 366 (94.1%) participants. Out of 23 who responded otherwise, 18 (78.26%) marked serology and the rest 5 (21.74%) considered chest x-ray as the standard diagnostic test.

Overall knowledge score related to COVID-19 was high in 220 (56.56%) participants while moderate and low in 165 (42.42%) and 4 (1.03%) participants respectively. No significant differences in knowledge on each question or cumulative knowledge score was observed among consultants and trainees (Table 1).

**Table 1:** Level of COVID-19 related knowledge among frontline physicians of Pakistan

	Consultants n (%)	Trainees n (%)	Total N(%)	***p***-value
**Among elderly, children, immuno-compromised and adults who do you think exhibit a high complication rate?**				
Score of 4	29 (27.4)	77 (72.6)	106 (100)	0.896
Score of below 4	71 (25.1)	212 (74.9)	283 (100)	
**What is mortality rate of COVID confirmed cases in Pakistan**				
Correct answer	93 (27.1)	250 (72.9)	343 (100)	0.083
Incorrect answer	7 (15.2)	36 (84.8)	46 (100)	
**Which of the following has higher yield for virus?**				
Correct answer	97 (26.4)	270 (73.6)	367 (100)	0.182
Incorrect answer	3 (13.6)	19 (86.4)	22 (100)	
**What is standard test for confirming a positive COVID case**				
Correct answer	95 (26)	271 (74)	366 (100)	0.654
Incorrect answer	5 (21.7)	18 (78.3)	23 (100)	
**Overall knowledge score**				
Low to moderate	39 (23.1)	130 (79.6)	169 (100)	0.298
High	61 (27.7)	159 (72.3)	220 (100)	

### Perception regarding the necessity of personal protective equipment in different care areas

Table 2 shows the distribution of various PPEs marked essential by frontline doctors in different clinical settings. Interestingly, highest number of participants marked gloves as the required PPE for all clinical areas followed by surgical mask, N-95 mask, full sleeves gown, eye shield and power air purifying respirators. Majority of participants marked N-95 mask as essential while taking nasopharyngeal samples and aerosol procedures (88%), assessing patients with respiratory symptoms (83%), in COVID patient care area (85%), ER triage (72%) and direct care of COVID-19 patient (82%). Almost half of them also believed that it should be used during surgical procedure of an asymptomatic patient.

**Table 2:** Perception regarding the necessity of personal protective equipment in different care areas

Patient care areas	Gloves n(%)	Surgical mask n(%)	N95 n(%)	Full-sleeves gown n(%)	Eye shield n(%)	PAPR n(%)
A	312 (80.2)	305 (78.4)	124 (31.9)	61 (15.7)	76 (19.5)	24 (6.2)
B	344 (88.4)	166 (42.7)	350 (90)	233 (59.9)	233 (59.9)	149 (38.3)
C	346 (88.9)	195 (50.1)	334 (85.9)	238 (61.2)	220 (56.6)	156 (40.1)
D	318 (81.7)	147 (37.8)	311 (79.9)	201 (51.7)	176 (45.2)	69 (17.7)
E	316 (81.2)	291 (74.8)	129 (33.2)	129 (33.2)	89 (22.9)	25 (6.4)
F	344 (88.4)	174 (44.7)	330 (84.8)	238 (61.2)	221 (56.8)	156 (40.1)
G	339 (87.1)	269 (69.2)	211 (54.2)	211 (54.2)	151 (38.8)	88 (22.6)
H	332 (85.3)	200 (51.4)	292 (75.1)	231 (59.4)	183 (47)	110 (28.3)

A = General patients area, B = Aerosol generating procedures and while taking nasopharyngeal swab, C = Patient area of COVID + case or suspect, D = During physical examination of patient with respiratory syndrome, E = During physical examination of patient without respiratory syndrome, F = Direct care of COVID + case, G = Surgical procedure on an asymptomatic patient, H = emergency triage, PAPR = powered air purifying respirator

### Prevalence of anxiety/depression and associated factors

Using a cut-off score of 8 or above, 166 (42.67%) participants were found to have anxiety/depression. Prevalence was significantly higher among younger physicians as compared to physicians more than 35 years of age (OR = 1.79, 95% CI: 1.19–2.69). Physicians working in emergency department were more likely to be depressed (OR = 3.50, 95% CI: 1.81–6.73) as compared to doctors working in clinics, wards and other units. Physicians who worked in hospitals where more than five COVID-19 patients were admitted were more likely to be depressed than those working in hospitals where five or less COVID-19 patients were admitted (OR = 2.17, 95% CI: 1.41–3.36). The frequency of anxiety/depression was also high among physicians who see more than five COVID suspects or confirmed cases every day (OR = 3.47, 95% CI: 2.4–5.40), are directly involved with COVID positive cases or suspects currently (OR = 2.73, 95% CI: 1.80–4.14), were working less than or equal to 20 h per week at the time of survey (OR = 1.78, 95% CI: 1.15–2.75), feared that they were unprotected (OR = 2.74, 95% CI: 0.87–5.24), had children at home (OR = 1.81, 95% CI: 1.21–2.72) and had moderate knowledge of the disease (OR = 3.83, 95% CI: 2.50–5.88). There was no association of gender, province, designation, working in public or private institution, presence of other adults and elderly members in the household and being satisfied with provision of PPEs. Only 12% of the doctors were fully satisfied with the provision of PPEs and almost 94% believed that they were completely unprotected or unprotected to some extent (Table 3).

**Table 3:** Distribution of participants’ characteristics and their univariable association with anxiety/depression

Variables	Anxious/Depressed n (%)	Non-anxious/non-depressed n (%)	Total N (%)	Crude OR (95% CI)	*p*-value
**Age (in years)**					
< 35	97 (49.7)	98 (50.3)	195 (100)	1.79 (1.19–2.69)	**0.005
≥ 35	69 (35.6)	125 (64.4)	194 (100)	Ref	
**Gender**					
Male	84 (41.8)	117 (58.2)	201 (100)	Ref	
Female	82 (43.6)	106 (56.4)	188 (100)	1.07 (0.72–1.61)	0.716
**Province**					
Sindh	79 (46.5)	91 (53.5)	170 (100)	1.16 (0.70–1.95)	0.562
Punjab	49 (37.7)	81 (62.3)	130 (100)	0.81 (0.47–1.41)	0.458
KPK + AJK + Balochistan	38 (42.7)	51 (57.3)	89 (100)	Ref	
**Designation**					
Residents	130 (45)	159 (55)	289 (100)	1.45 (0.91–2.32)	0.118
Consultants	36 (36)	64 (64)	100 (100)	Ref	
**Current area of practice**					
Public sector	97 (46.6)	111 (53.4)	208 (100)	1.42 (0.94–2.13)	0.091
Private sector	69 (38.1)	112 (61.9)	181 (100)	Ref	
**Current area of work**					
Emergency	87 (59.2)	60 (40.8)	147 (100)	3.50 (1.81–6.73)	** < 0.001
OPD	50 (32.7)	103 (67.3)	153 (100)	1.17 (0.61–2.26)	0.639
Ward	12 (38.7)	19 (61.3)	31 (100)	1.52 (0.61–3.81)	0.369
Other	17 (29.3)	41 (70.7)	58 (100)	Ref	
**What is the mortality rate among those who are positive in Pakistan?**					
≤ 5%	146 (42.6)	197 (57.4)	343 (100)	0.96 (0.52–1.79)	0.906
> 5%	20 (43.5)	26 (56.5)	46 (100)	Ref	
**Are you at present directly involved in care of COVID confirmed patients?**					
No	59 (30.6)	134 (69.4)	193 (100)	Ref	
Yes	107 (54.6)	89 (45.4)	196 (100)	2.73 (1.80–4.14)	** < 0.001
**How many patients with COVID are admitted in your hospital?**					
≤ 5	98 (36.7)	169 (63.3)	267 (100)	Ref	
> 5	68 (55.7)	54 (44.3)	122 (100)	2.17 (1.41–3.36)	** < 0.001
**How many COVID suspects do you see every day?**					
≤ 5	85 (32.7)	175 (67.3)	260 (100)	Ref	
> 5	81 (62.8)	48 (37.2)	129 (100)	3.47 (2.24–5.40)	** < 0.001
**How many hours/ week are you working?**					
≤ 20 h	62 (52.5)	56 (47.5)	118 (100)	1.78 (1.15–2.75)	*0.010
> 20 h	104 (38.4)	167 (61.6)	271 (100)	Ref	
**Do you fear that you are unprotected?**					
No	8 (33.3)	16 (66.7)	24 (100)	Ref	
To some extent	64 (35)	119 (65)	183 (100)	1.08 (0.44–2.65)	0.874
Yes	94 (51.6)	88 (48.4)	182 (100)	2.14 (0.87–5.24)	0.097
**Are you satisfied with the personal protective equipment provided to you?**					
No	66 (45.2)	80 (54.8)	146 (100)	1.02 (0.53–1.98)	0.950
To some extent	79 (40.3)	117 (59.7)	196 (100)	0.84 (0.44–1.59)	0.584
Yes	21 (44.7)	26 (55.3)	47 (100)	Ref	
**Your household members include adults**					
No	59 (41.8)	82 (58.2)	141 (100)	Ref	
Yes	107 (43.1)	141 (56.9)	248 (100)	1.06 (0.69–1.60)	0.803
**Your household members include elderly**					
No	101 (40.1)	151 (59.9)	252 (100)	Ref	
Yes	65 (47.4)	72 (52.6)	137 (100)	1.35 (0.89–2.05)	0.161
**Your household members include children**					
No	70 (35.5)	127 (64.5)	197 (100)	Ref	
Yes	96 (50)	96 (50)	192 (100)	1.81 (1.21–2.72)	**0.004
**Overall knowledge of COVID-19**					
Moderate to low	103 (60.9%)	66 (39.1)	169 (100)	3.89 (2.54–5.96)	** < 0.001
High	63 (28.6)	157 (71.4)	220 (100)	Ref	

*KPK* khyber pakhtunkhwa, *AJK* Azad Jammu & Kashmir, *Ref* reference category set in logistic regression model, *OR* odd ratio, *CI* confidence interval

**significant at *p* < 0.01, * significant at *p* < 0.05

Final multivariate logistic regression model was developed with age, designation, current practice area, current area of work, no. of COVID suspects seen per day, working hours per week, fear of non-protection, presence of elderly members and children at home and overall knowledge score. After controlling the effects of age, designation, working in public or private sector, current area of practice and fear of being unprotected, the odds of anxiety/depression was high in physicians who were assessing more than five COVID suspects or patients per day (aOR = 2.73, 95% CI: 1.65–4.52), who were working than 20 h/week or less (aOR = 2.11, 1.27–3.49), had children among household members (aOR = 1.58, 95% CI: 1.00–2.50) and had moderate to low knowledge of the infection (aOR = 2.69, 95% CI: 1.68–4.31) (Table 4).

**Table 4:** Association of factors with depression on final multivariable binary logistic regression model

	Adjusted OR	95% CI	***P***-value
**Age (in years)**			
< 35	1.12	0.67–1.89	0.670
≥ 35	Ref		
**Designation**			
Trainee	1.43	0.79–2.56	0.236
Consultant	Ref		
**Current area of practice**			
Public sector	1.21	0.75–1.97	0.432
Private sector	Ref		
**Current area of work**			
Emergency	1.86	0.87–3.97	0.111
OPD	1.07	0.53–2.18	0.854
Ward	1.08	0.38–3.06	0.880
Other	Ref		
**No. of COVID suspects/confirmed cases seen per day**			
≤ 5	Ref		
> 5	2.73	1.65–4.52	** < 0.001
**Working hours per week**			
≤ 20 h	2.11	1.27–3.49	** < 0.004
> 20 h	Ref		
**Household members include children**			
Yes	1.58	1.00–2.50	*0.049
No	Ref		
**Fear of non-protection**			
Yes	1.40	0.532–3.69	0.494
To some extent	0.89	0.34–2.34	0.891
No	Ref		
**Overall level of knowledge related to COVID-19**			
Moderate to low	2.69	1.68–4.31	** < 0.001
High	Ref		

*Ref* reference category, *OR* odds ratio, *CI* confidence interval

**significant at *p* < 0.01, *significant at *p* < 0.05

## Discussion

This study explores the affect of COVID-19 pandemic on the mental health of frontline physicians in Pakistan. Almost equal number of male and female doctors participated and the response was highest from provinces with the highest number of reported COVID-19 cases in Pakistan. Both consultants and trainees working in emergency, clinics, ward and other hospital services at the time of survey participated in the study.

This study found a 43% prevalence of anxiety/depression among doctors within a month of detection of the first case of COVID-19, which says a lot about the upcoming storm of mental health issues among the group. Lai J et al. in March 2020 reported a frequency of up to 40% of mild to moderate depressive symptoms among doctors of China during the outbreak [9], while another survey on a small number of health care workers in China found that the workers showed signs of psychological distress during the pandemic [15].

Though data assessing the effect of the recent pandemic on mental health of doctors is sparse especially in the developing countries, but a previous survey in 2016 among doctors working in a tertiary care hospital in Pakistan reported an association of female gender and more service years with anxiety and depression [12]. Even among the general population depression and anxiety related disorders are found to be more common among females [16]. This contrasts with findings of our study in which although equal number of males and females participated, yet gender was not associated with anxiety/depression. Moreover, physicians who were less than 35 years of age were more likely to be depressed than older doctors. Having children at home was another factor associated with anxiety and depression; and younger doctors were more likely to have children at home and hence more likely to be anxious about taking infection to their loved ones at home and hence distressed.

There was no significant difference in the frequency of anxiety/depression among trainees and consultants, yet a moderate to less knowledge score was more likely to be associated with anxiety/depression. Interestingly, the knowledge scores were not different among trainees and consultants probably because COVID-19 is a new disease and the experiential knowledge about the pandemic is likely to be same among trainees and consultants. Therefore, even after controlling for age and designation, low to moderate scores were associated with anxiety/depression in the multivariable model.

Fear of being unprotected was another factor associated with anxiety/depression in the univariate analysis, which is plausible. The healthcare system in Pakistan is weak and may not able to able to cope with the overwhelming burden of the pandemic. Infection control practices and availability of PPE is also sub-optimal in most of the hospitals. Due to a sudden and exponential rise of positive cases and patients needing medical care, even in developed countries such as USA and UK, PPE is not readily available because of which a high number of the healthcare workers have already been infected [4]. Another study in China among health workers during this pandemic also reported fear among the staff for shortage of PPE [15]. Though the Center for Disease Control and Prevention (CDC) recommends that during crisis situations, N-95 respirator masks be used only during aerosol-generating procedures [17], in our study more than 80% of the doctors thought that N-95 mask should be available not only during aerosol generating procedures but while examining patients with respiratory symptoms, in emergency care, in areas where there are COVID-19 suspects and while caring for confirmed cases. This explains majority (94%) of the frontline doctors feeling unprotected completely or to some extent and hence more likely to have anxiety and depression.

In the univariable analysis, doctors working in emergency and those directly in contact with COVID suspects and positive cases were more likely to be depressed as this group is most exposed and hence anxious. This is in line with a recent study conducted in China where frontline health workers engaged in direct diagnosis, treatment, and care of patients with COVID-19 were at a higher risk of symptoms of anxiety/depression [9]. Being in contact with more than five COVID confirmed cases /day was associated with anxiety and depression both in univariable and multivariable analysis.

Interestingly, working less than 20 h a week was positively associated with anxiety/depression both in univariable and multivariable analysis, which is in contrast with previous studies reporting association of long working hours among trainees and doctors with poor mental health. In a study in Japan, working for more than 80 h per week was associated with a significantly higher risk of developing depression [18]. Another study in UK showed association of more than 70 working hours per week with depression among young graduates [19]. A plausible explanation of this finding is that a complete lock down and social distancing was being observed in the country when this survey was carried out. Moreover, patients were encouraged to stay at home if symptoms are not severe, hence frontline doctors may have less duties to attend. It is already known that social network structure and function are strongly intertwined with anxiety and depressive symptoms [12], therefore it is possible that doctors who are working less than 20 h a week are more likely to be distressed due to unusually less work and less social interaction. This may be compounded by feelings of guilt of leaving colleagues to work instead of them [20].

Moreover, lock down and social distancing may have a negative impact on psychosocial well being generally and more research needs to be done to explore the effects of social distancing on the mental health of general population.

To the best of our knowledge this was the first study to determine the factors associated with anxiety and depression among a representative and diverse group of frontline physicians from 65 cities of Pakistan. Yet, depression and anxiety may have been underreported here as mental illness is a social stigma in our part of the world not only among the general population but also among healthcare providers [21, 22]. Moreover, we only used one scale to report depression and anxiety as the survey was online and asking a too many questions from frontline doctors was not feasible. Moreover, a possibility of participation bias in online surveys cannot be excluded, where the potential participants who are suffering from anxiety and depression do not choose to participate in the study. This would also result in an underreporting of the prevalence of anxiety/depression among frontline physicians.

## Conclusion

This study reports 43% prevalence of anxiety/depression among frontline physicians of Pakistan within a span of just one month of diagnosis of the first positive case and hence rationalizes the need to address mental health and wellbeing of doctors caring for patients during this pandemic. On 27th April, The New York Times reported suicide of frontline doctor working in emergency department. This is indeed a devastating news for the medical fraternity globally [23]. Factors associated with psychological distress which lead to symptoms of anxiety, depression and hence provoke suicidal ideation should be explored and efforts made to control modifiable factors. Doctors need to be provided adequate PPEs with optimum infection control practices so that they feel protected and safe. Hospital management needs to take their doctors in confidence and make sure that the doctors are provided with medical coverage for themselves and their families in order to reduce their work stress and insecurities. Adapted psychological interventions such as online counseling and cognitive behavioral therapy by trained psychologists to support mental health of physicians is recommended. Moreover, assessing the effectiveness of such interventions on psychological wellbeing of frontline health workers during this pandemic would be worthwhile.

## Supplementary information

**Additional file 1.** Knowledge Questions.

## Data Availability

The data analyzed in the current study is available in Google drive that can be accessed on request from corresponding author.

## Abbreviations

CDC: Center for disease control and prevention
CI: Confidence interval
COVID-19: Coronavirus disease of 2019
ER: Emergency
IQR: Inter-quartile range
OPD: Out-patient department
OR: Odd ratio
aOR: Adjusted odd ratio
PPE: Personal protective equipment
SRQ: Self-reported questionnaire
UK: United Kingdom
USA: United Stated of America
