# Supplementary material for: COVID-19 pandemic- knowledge, perception, anxiety and depression among frontline doctors of Pakistan
Source: BMC Psychiatry. 2020 Sep 23;20:459. doi: 10.1186/s12888-020-02864-x (PMC7509498; doi:10.1186/s12888-020-02864-x)
Supplement: Supplementary file 1 — Additional file 1. Knowledge Questions. [file 12888_2020_2864_MOESM1_ESM.docx]

**Appendix A**

**Knowledge Questions**

| **Questions** | **Correct answers** |
| --- | --- |
| **Which of the following exhibit a high complication rate?** | |
| Elderly Yes No | Yes |
| Children Yes No | No |
| Immuno-compromised Yes No | Yes |
| Adults Yes No | No |
| **What is the mortality rate of COVID-19 confirmed cases in Pakistan?** | |
| ≤5% 6-10% >10% | 5% |
| **Which of the following has higher yield for virus?** |  |
| Bronchoalveolar lavage Sputum Nasopharyngeal swab  Blood | bronchoalveolar lavage and nasopharyngeal swab |
| **What is standard test for confirming a positive COVID case** | |
| Serology Chest X-ray CT Chest PCR test | PCR test |
